# Prevalence and Characteristics of Probable Major Depression and Bipolar Disorder within UK Biobank: Cross-Sectional Study of 172,751 Participants

**DOI:** 10.1371/journal.pone.0075362

**Published:** 2013-11-25

**Authors:** Daniel J. Smith, Barbara I. Nicholl, Breda Cullen, Daniel Martin, Zia Ul-Haq, Jonathan Evans, Jason M. R. Gill, Beverly Roberts, John Gallacher, Daniel Mackay, Matthew Hotopf, Ian Deary, Nick Craddock, Jill P. Pell

**Affiliations:** 1 Institute of Health and Wellbeing, Mental Health and Wellbeing Research Group, University of Glasgow, Glasgow, United Kingdom; 2 Centre for Cognitive Ageing and Cognitive Epidemiology, Department of Psychology, University of Edinburgh, Edinburgh, United Kingdom; 3 Institute of Psychiatry, Kings College London, London, United Kingdom; 4 National Centre for Mental Health, Institute of Neurosciences and Mental Health, Cardiff University, Cardiff, United Kingdom; 5 Institute of Health and Wellbeing, General Practice and Primary Care, University of Glasgow, Glasgow, United Kingdom; 6 Institute of Health and Wellbeing, Public Health, University of Glasgow, Glasgow, United Kingdom; University of Iowa Hospitals & Clinics, United States of America

## Abstract

**Objectives:**

UK Biobank is a landmark cohort of over 500,000 participants which will be used to investigate genetic and non-genetic risk factors for a wide range of adverse health outcomes. This is the first study to systematically assess the prevalence and validity of proposed criteria for probable mood disorders within the cohort (major depression and bipolar disorder).

**Methods:**

This was a descriptive epidemiological study of 172,751 individuals assessed for a lifetime history of mood disorder in relation to a range of demographic, social, lifestyle, personality and health-related factors. The main outcomes were prevalence of a probable lifetime (single) episode of major depression, probable recurrent major depressive disorder (moderate), probable recurrent major depressive disorder (severe), probable bipolar disorder and no history of mood disorder (comparison group). Outcomes were compared on age, gender, ethnicity, socioeconomic status, educational attainment, functioning, self-reported health status, current depressive symptoms, neuroticism score, smoking status and alcohol use.

**Results:**

Prevalence rates for probable single lifetime episode of major depression (6.4%), probable recurrent major depression (moderate) (12.2%), probable recurrent major depression (severe) (7.2%) and probable bipolar disorder (1.3%) were comparable to those found in other population studies. The proposed diagnostic criteria have promising validity, with a gradient in evidence from no mood disorder through major depression and probable bipolar disorder in terms of gender distribution, socioeconomic status, self-reported health rating, current depressive symptoms and smoking.

**Significance:**

The validity of our proposed criteria for probable major depression and probable bipolar disorder within this cohort are supported by these cross-sectional analyses. Our findings are likely to prove useful as a framework for a wide range of future genetic and non-genetic studies.

## Introduction

The UK Biobank cohort was recently launched as a landmark resource for health-related research which is available to researchers across the world [1]. It comprises more than 500,000 men and women aged 40–69 years recruited from the general population in the United Kingdom and who represent a wide range of exposures typical of the UK population [2]. Baseline assessments were extensive [2]. Prospective waves of follow-up, along with linkage to routine National Health Service (NHS) information will, in due course, allow a comprehensive evaluation of the contribution of genetics, lifestyle, diet and aspects of the environment to a wide range of health outcomes, including diabetes, cardiovascular disease, cancer, dementia and death. During the last two years of recruitment, questions on depressive and manic symptoms were added to the assessment protocol (n = 172,751 participants) [3].

Relative to other areas of medicine, large-scale population cohorts that have been assessed in detail with respect to mood disorder features are scarce, even though the public health and economic impact of these conditions is considerable [4], [5]. The UK Biobank cohort therefore represents a valuable opportunity to study the long-term impact of mood disorder symptoms on a wide range of health and cognitive outcomes. There will also be opportunities to integrate genetic and environmental data to better understand risk for mood disorder, as well as comorbidity with many physical disorders such as obesity and cardiovascular disease and, longer-term, risk for dementia and premature mortality.

Here we present criteria agreed by our mental health working-group for classifying a *probable* lifetime history of mood disorder (both major depression and bipolar disorder) using the information collected by UK Biobank on these 172,751 participants. We then use the baseline data in a descriptive epidemiological study to compare the demographic, lifestyle and personality characteristics of participants according to whether they meet the criteria and, thereby, provide some evidence towards the likely validity of these criteria.

We anticipate that this work will facilitate future genetic and non-genetic research on mood disorder within the cohort and provide a framework for defining mood disorder within large population cohorts of this kind.

## Materials and Methods

**Figure 1:**
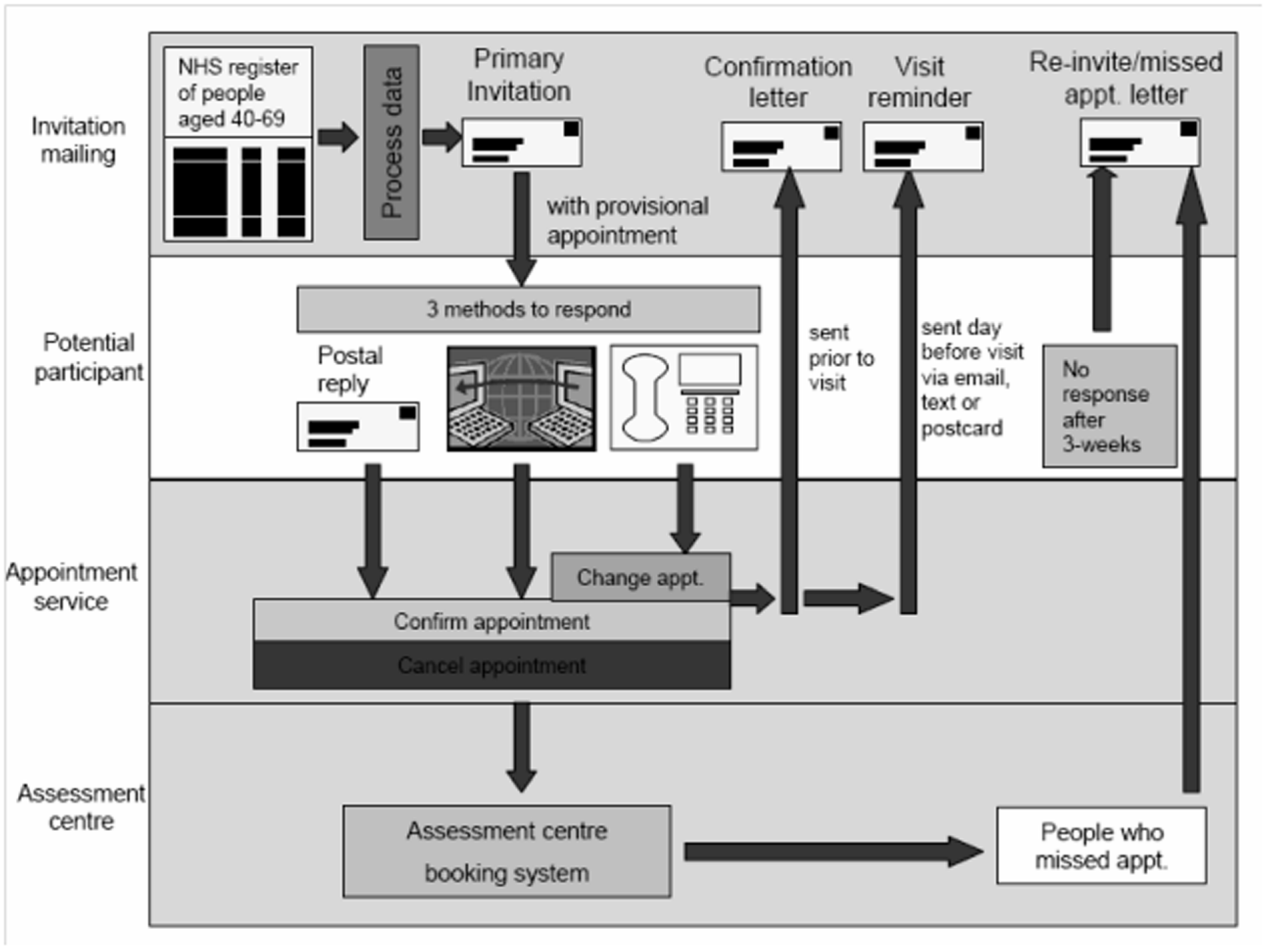
Invitation and recruitment to UK Biobank.

UK Biobank invitations and recruitment occurred during 2006–2010. More than 500,000 men and women aged 40–69 years were assessed, having been identified from NHS patient registers and as living within a reasonable travelling distance of an assessment centre (Figure 1). In the final two years of recruitment 172,751 participants were assessed at baseline with respect to depressive and manic symptoms. These assessments were part of an extensive touch-screen questionnaire, more details of which are available on the UK Biobank website [3].

### Defining major depression and bipolar disorder

**Figure 2:**
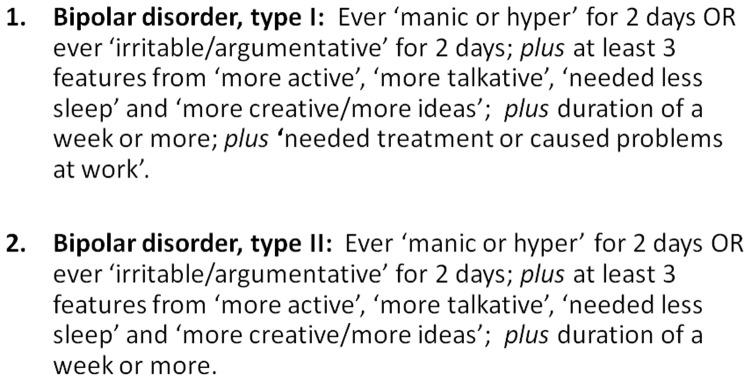
Proposed criteria for lifetime experience of probable bipolar disorder (type I and type II).

Questions for assessing a probable history of probable bipolar disorder were based on the approach for manic symptoms within the Structured Clinical Interview for DSM-IV Axis I Disorders (SCID-I) [6]. These questions were suggested by DJS, NC and JG and approved by the UK Biobank steering committee (Figure 2) The questions were: *"Have you ever had a period of time lasting at least two days when you were feeling so good, "high", excited or "hyper" that other people thought you were not your normal self or you were so "hyper" that you got into trouble?"; "Have you ever had a period of time lasting at least two days when you were so irritable that you found yourself shouting at people or starting fights or arguments?"; "Please try to remember a period when you were in a "high" or "irritable" state and select which of the following apply: I was more active than usual, I was more talkative than usual, I needed less sleep than usual, I was more creative or had more ideas than usual."*

**Figure 3:**
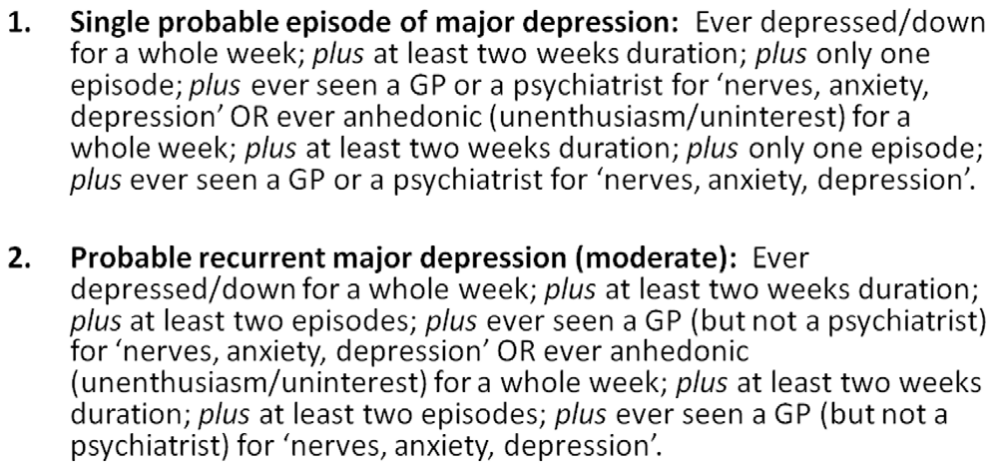
Proposed criteria for lifetime experience of major depressive disorder.

Current and previous depressive symptoms were assessed by items relating to the lifetime experience of minor and major depression [3], items from the Patient Health Questionnaire (PHQ) [7] and items on help-seeking for mental health (Figure 3): *"Looking back over your life, have you ever had a time when you were feeling depressed or down for at least a whole week?"; "Have you ever had a period of time lasting at least two days when you were so irritable that you found yourself shouting at people or starting fights or arguments?"; "How many weeks was the longest period when you were feeling depressed or down?"; "How many periods have you had when you were feeling depressed or down for at least a whole week?"; "Have you ever seen a general practitioner (GP) for nerves, anxiety, tension or depression?"; "Have you ever seen a psychiatrist for nerves, anxiety, tension or depression?"*

Neuroticism, a depression-related personality trait, was assessed using 12 questions from the Eysenck Personality Inventory Neuroticism scale (EPIN-R) [3], [8]: *"Does your mood often go up and down?"; "Do you ever feel 'just miserable' for no reason?"; "Are you an irritable person?”; "Are your feelings easily hurt?"; "Do you often feel 'fed-up'?"; "Would you call yourself a nervous person?"; "Are you a worrier?"; "Would you call yourself tense or 'highly strung'?"; "Do you worry too long after an embarrassing experience?"; "Do you suffer from 'nerves'?"; "Do you often feel lonely?"; "Are you often troubled by feelings of guilt?"*

We convened a series of meetings of Biobank-approved researchers focusing on mental health and cognition (membership DJS, JPP, DM, NC, JG, MH, BC, BN, DM, JE, ID and BR) and after a number of iterations of proposed diagnostic criteria we agreed the definitions for classifying probable bipolar disorder and probable major depression, as outlined in Figures 2 and 3 respectively (Unique Data Identifier (UDI) codes listed within Appendix S1). This included a spectrum of severity from probable single lifetime episode of major depression, through probable recurrent major depression (moderate), probable recurrent major depression (severe) and probable bipolar disorder (types I and II). In order to identify these clinically meaningful mood disordered and non-mood disordered groups, we did not include participants with mild manic symptoms (Figure 2 criteria with less than one week duration and no treatment/problems) or mild depressive symptoms (Figure 3 criteria but not assessed by a health professional). Although as noted above neither depressive nor manic symptoms were assessed according to a precise diagnostic schedule, as far as possible we followed the structured diagnostic approach within the International Classification of Diseases (ICD-10) [9] and the American Psychiatric Association's Diagnostic and Statistical Manual (DSM-IV) [10] (Figure 2 and Figure 3).

### Towards the validity of proposed criteria for major depression and bipolar disorder

To investigate the validity of our proposed definitions of probable major depression and probable bipolar disorder, we used available data on social, demographic, lifestyle, personality and clinical variables from the UK Biobank baseline assessment and compared across the mood disorder spectrum, from a ‘no mood disorder’ comparison group, through probable single episode major depression, probable recurrent major depression (moderate), probable recurrent major depression (severe) and probable bipolar disorder.

The variables included in these analyses were: gender; ethnicity; socioeconomic status (neighbourhood-level, assessed using the Townsend deprivation score where a negative score represents greater affluence [11]); educational level (age left education and whether achieved an undergraduate degree); self-reported inability to work due to sickness; self-assessment of having a long-term illness; self-assessment of overall health rating; current depressive symptoms (defined as ‘depressed mood’ and/or ‘unenthusiasm/uninterest’ (anhedonia) for at least ‘nearly every day in past 2 weeks’); neuroticism score; smoking status and alcohol use.

All descriptive analyses were carried out using the statistical software Stata [12] and SPSS [13]. In order to examine the association between probable mood disorder category and demographic and clinical variables, tests of association were used which accounted for trend across the categories. An extension of the Kruskal-Wallis test allowed for the comparison of both continuous and categorical variables across an ordered categorical variable (*nptrend* command in Stata) [14].

This study was conducted under generic approval from the NHS National Research Ethics Service (approval letter dated 17^th^ June 2011, Ref 11/NW/0382).

## Results

### Prevalence of a lifetime diagnosis of probable major depression and bipolar disorder

**Figure 4:**
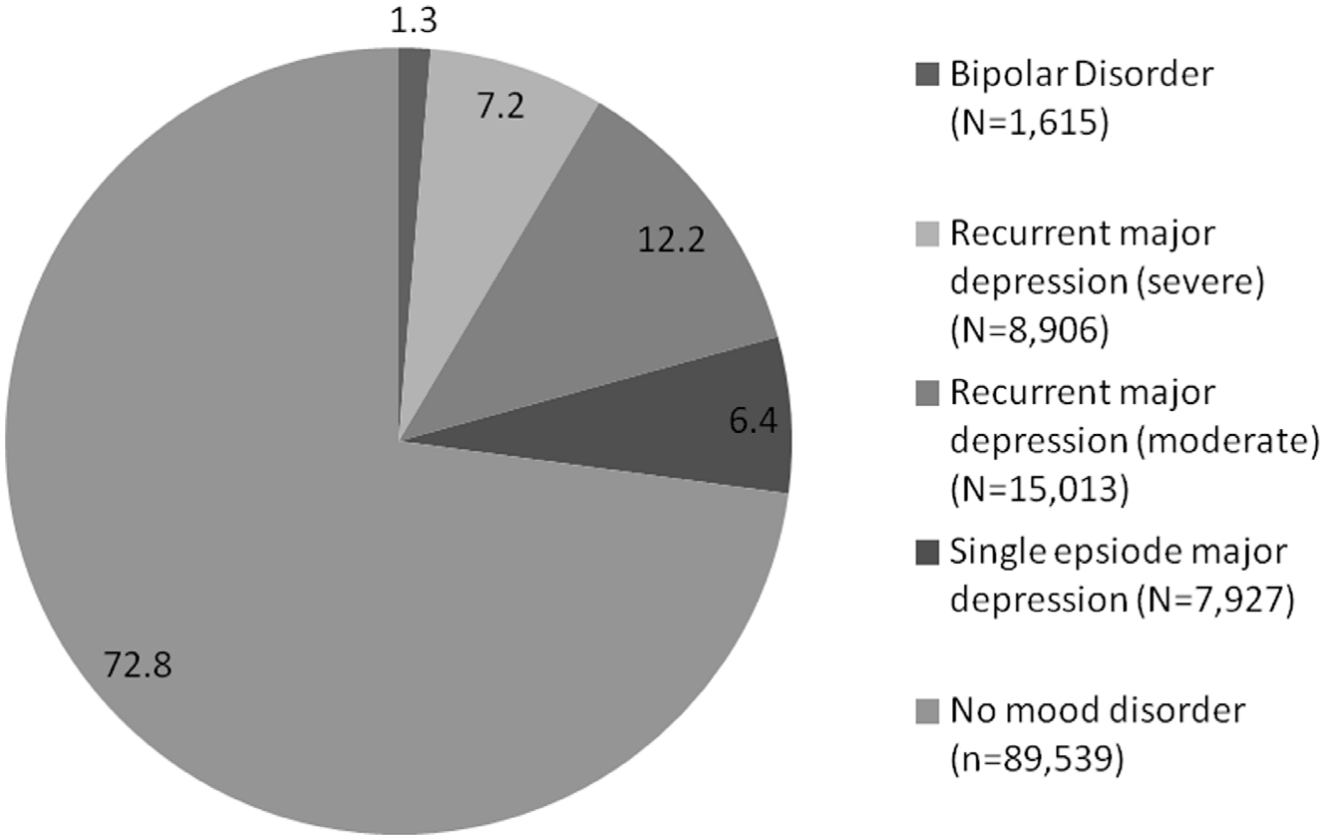
Percentage diagnostic breakdown of probable lifetime diagnoses of mood disorder (n = 123,000).

From the sample of 172,751 participants who were assessed for depressive and manic symptoms, 149,847 provided sufficient data to allow an assessment of probable bipolar disorder and/or probable major depression. From these, 26,847 did not meet our diagnostic criteria for a mood disorder but were excluded because of sub-threshold manic or depressive symptoms. The remaining 123,000 participants were coded within mood disordered and non-mood disordered groups as follows: 7,927 (6.4% satisfied criteria for a probable single lifetime episode of major depression; 15,013 (12.2%) for probable recurrent major depression (moderate); 8,906 (7.2%) for probable recurrent major depression (severe); 1,615 (1.3%) for probable bipolar disorder (types I and II combined; bipolar I, n = 808 and bipolar II, n = 807); and 89,539 (72.8%) participants did not satisfy any of our proposed mood disorder criteria and formed a ‘no mood disorder’ comparison group (Figure 4).

### Information on validity of proposed criteria for probable major depression and bipolar disorder

Most of the demographic features we assessed provided some support for the validity of our proposed criteria (Table 1). Given the large sample sizes, comparisons across groups were statistically significant (p<0.001) for all variables except ‘age left education’. However, for certain variables the magnitude of these differences was small (Table 1).

**Table 1:** Demographic and clinical characteristics (N = 123,000)

	Bipolar disorder N = 1,615	Recurrent major depression (severe) N = 8,906	Recurrent major depression (moderate) N = 15,013	Single episode major depression N = 7,927	No mood disorder N = 89,539
Age *Mean (SD)*	54.5 (8.08)	55.6 (8.05)	55.4 (7.93)	56.3 (8.01)	57.4 (8.11)
Female *N (%)*	790 (48.9)	5145 (57.8)	10325 (68.8)	5042 (63.6)	44678 (49.9)
White ethnicity *N (%)*	1422 (88.8)	8232 (92.8)	14176 (94.7)	7575 (96.0)	81566 (91.5)
Townsend deprivation score *Mean (SD)*	0.06 (3.31)	−0.47 (3.18)	−1.08 (2.95)	−1.35 (2.77)	−1.31 (2.87)
Age left education *Mean (SD)*	16.9 (2.73)	16.8 (2.70)	16.8 (2.34)	16.9 (2.35)	16.8 (2.46)
Undergraduate degree *N (%)*	578 (36.0)	3205 (36.2)	5211 (34.9)	2893 (36.7)	29365 (33.1)
Unable to work due to sickness *N (%)*	228 (14.2)	1038 (11.7)	678 (4.5)	199 (2.5)	1799 (2.0)
Long-term illness *N (%)*	912 (58.5)	4639 (53.3)	5845 (39.8)	2650 (34.0)	24773 (28.2)
Overall health rating poor *N (%)*	226 (14.1)	1222 (13.8)	1058 (7.1)	294 (3.7)	2645 (3.0)
Current depressive symptoms *N (%)*	194 (12.2)	876 (9.9)	675 (4.5)	135 (1.7)	1359 (1.5)
Neuroticism score *Mean (SD)*	6.57 (3.58)	6.82 (3.39)	5.79 (3.18)	4.15 (3.00)	3.26 (2.91)
Current smoker *N (%)*	286 (17.8)	1139 (12.8)	1279 (8.5)	621 (7.9)	5256 (5.9)
Daily/almost daily alcohol *N (%)*	326 (20.2)	1773 (19.9)	2896 (19.3)	1616 (20.4)	18762 (21.0)

#### Gender distribution

The male to female ratio for the probable bipolar disorder group was approximately equal whereas there was a predominance of women in the probable recurrent depression and probable single episode depression groups. For probable recurrent major depression (moderate) the ratio of females to males was approximately 7∶3.

#### Ethnicity

There were differences across the mood disorder groups and the comparison group with respect to ethnicity. The probable bipolar disorder group had a significantly higher proportion of non-white individuals, accounted for mostly by a higher proportion of people who described their ethnicity as either Asian/Asian British or Black/Black British: 7.4% of the probable bipolar group compared to 3.3% in the probable recurrent depression (moderate) group, 2.6% in the probable single episode depression group, and 6.4% in the no mood disorder comparison group.

#### Socioeconomic status

The probable bipolar disorder group had a significantly higher (more deprived) Townsend score and there was a gradient from more to less affluent as severity of mood disorder increased.

#### Educational level

The four probable mood disordered groups had a greater proportion of individuals who had achieved an undergraduate degree (between 35-37% versus 33% in the non-mood disordered comparison group). Among those who did not have a degree, the mean age of leaving full-time education was approximately the same across all groups (16.8 or 16.9 years).

#### Self-reported functional impairment and ratings of health

There was a clear gradient in terms of the proportion of individuals in each group who rated themselves as unable to work due to sickness. In the probable bipolar disorder group this figure was 14.2%, versus 11.7% in probable recurrent depression (severe) group, 4.5% in the probable recurrent depression (moderate) group, 2.5% in the probable single episode depression group and 2.0% in the comparison group. Similarly, self-reported long-term illness levels were highest in the probable bipolar disorder group (58.5%), lowest in the comparison group (28.2%) and at intermediate levels in the probable depressive disorder groups.

This gradient was also a feature in terms of the proportion in each group who described their overall health rating as poor (14.1% in the probable bipolar disorder group, 13.8% in the probable recurrent depression (severe) group, 7.1% in the probable recurrent depression (moderate) group, 3.7% in the probable single episode depression group and 3.0% in the no mood disorder comparison group).

#### Current depressive symptoms and neuroticism scores

The proportion of individuals with current depressive symptoms (defined as depressed mood and/or anhedonia almost every day for the preceding 2 weeks) was significantly different across the groups. The probable bipolar disorder group had the highest levels of current depressive symptoms (12.2%), followed by the probable recurrent depression (severe) group (9.9%), then the probable recurrent depression (moderate) group (4.5%), the probable single episode depression group (1.7%) and the no mood disorder comparison group (1.5%).

Similarly, mean neuroticism scores were highest in the probable bipolar disorder and probable severe depression groups (6.57 and 6.82 respectively), lowest in the probable single episode depression (4.15) and no mood disorder (3.26) groups and at an intermediate level in the probable moderate recurrent depression group (5.79).

#### Smoking status and alcohol use

Current smoking rates were highest in the probable bipolar disorder group at 17.8% and lowest in the no mood disorder comparison group (5.9%), with a gradient of higher smoking rates as severity of depression increased.

No such association was observed for self-reported daily/almost daily alcohol use, which was similar across all groups at approximately 20%. However, those within the severe depression and probable bipolar disorder groups were more likely than the other groups to report that they did not currently drink alcohol but had done previously (62.2% for probable severe depression and 58.9% for probable bipolar disorder, compared to 37.6% in the no mood disorder comparison group) and when asked for a reason for stopping alcohol, 34.3% of the probable severe depression group and 32.3% of the probable bipolar disorder group cited ‘illness or ill health’ compared to 19.3% in the comparison group.

## Discussion

Our goal was to propose a set of criteria for a probable lifetime history of major depression or bipolar disorder - within the confines of the assessment questions which were used - and then provide some evidence towards the likely validity of these criteria. Overall, the lifetime prevalence rates of probable bipolar disorder, probable recurrent major depression and probable single episode major depression were consistent with other population based lifetime estimates (1.3% for bipolar disorder and 25.8% for major depression) [15], [16]. However, it should be noted that our reliance on participant self-report of symptoms, and our use of criteria that are likely to be somewhat less stringent than formal diagnostic criteria, mean that these estimates of prevalence are for *probable* lifetime diagnoses only, rather than actual or confirmed diagnoses. The majority of the demographic and clinical variables we compared provided some support for the validity of our proposed diagnostic criteria. For example, the gender distributions of approximately 1∶1 for probable bipolar disorder and approximately 2∶1 (female∶male) for probable recurrent depression are consistent with a large body of epidemiological research on lifetime rates of mood disorder in males and females [15], [17]–[21].

As might be expected, self-reported rates of inability to work due to sickness, of long-term illness and of poor overall health were highest in the probable bipolar disorder and probable severe depression groups and lowest in the probable single episode depression and comparison groups, but perhaps most convincing in terms of these comparisons were findings with respect to current depressive symptoms and neuroticism scores. For depressive symptoms, 12.2% of the probable bipolar disorder group satisfied our criteria for current depression versus 9.9% for the probable severe recurrent depression group, 4.5% for the probable moderate recurrent depression group, 1.7% for the probable single episode depression group and 1.5% for the no mood disorder comparison group. This is consistent with several longitudinal studies of bipolar disorder which have identified that the experience of clinically significant (although often sub-diagnostic) depressive symptoms is very common [22]–[24].

For neuroticism scores, both the probable bipolar disorder and the probable severe depression groups scored highest (6.57 and 6.82 respectively), the other depression groups were lower, and the comparison group was lowest. Neuroticism is widely accepted as a vulnerability marker for a range of mental health problems but particularly mood disorders [25]–[30] and there is a recognised genetic correlation between neuroticism and mood states [31], [32]. This finding provides support for the validity of our mood disorder definitions, even though current low mood may have to some extent raised scores on the EPI.

For socioeconomic status, there was a gradient from more to less affluent as severity of mood disorder increased (from no mood disorder to probable bipolar disorder), in keeping with some studies linking more deprived social circumstances with risk of severe mental disorder [33]–[35]. However, our findings for ethnicity and educational attainment did not show such a clear gradient across the groups. Although the probable bipolar disorder group had a higher proportion of individuals who described their ethnicity as Asian/Asian British or Black/Black British (7.4%) - compared to the probable recurrent depression (moderate) group (3.3%) and the probable single episode depression group (2.6%) - this figure was close to the figure of 6.4% for the comparison group. Similarly, the mean age of leaving full-time education and the percentage of individuals in each group with an undergraduate degree was broadly similar across mood disorder groups (35-37% compared to 33.1% for the non-mood disordered comparison group). It is possible that this finding represents a recall bias, with better remembering of depressive episodes in those with a stronger educational background.

Although current smoking rates were highest in the probable bipolar disorder and probable severe depression groups (17.8% and 12.8% respectively) and lowest in controls (5.9%), these rates are somewhat lower than might be expected across all groups, suggesting a recruitment bias towards non-smokers within the cohort as a whole. In general, smoking rates in major mental illnesses are recognised to be much higher than in the general population (50–70% compared to 20%–30%) [36].

We did not find a substantial difference between the mood disordered groups and controls in terms of the proportion reporting daily or almost daily alcohol use (approximately 20% across all groups) but more detailed questions about previous alcohol use suggested that a larger proportion of individuals in the probable bipolar disorder and probable severe depression groups had stopped using alcohol for health reasons. This is in keeping with epidemiological research on the high comorbidity between severe mood problems such as probable bipolar disorder and alcohol use disorders. For example, the Epidemiologic Catchment Area (ECA) study in the United States found that individuals with bipolar I disorder (BPI) had a lifetime prevalence rate of alcohol abuse or dependence of 46.2% compared to 16.5% for individuals with major depressive disorder [37]. Similarly, the National Comorbidity Survey Replication Study identified a lifetime comorbidity between bipolar I disorder and alcohol abuse of 56.3% [20].

### Strengths and Weaknesses

This is a very large population-based cohort of individuals in middle age recruited from diverse backgrounds across the UK. We have attempted to devise and test criteria for a probable lifetime history of major depression or bipolar disorder which are clinically meaningful, for example, by specifying minimum duration and severity criteria, as well as including impairment of functioning criteria (Figures 2 and 3). It should be noted that the criteria for depression were based largely only on low mood and anhedonia as core symptoms and that data on additional depressive symptoms were not available.

The boundaries of normality, major depression and bipolar disorder remain a topic of considerable debate and, as such, any diagnostic criteria for these conditions can only be considered proposals which will require further clinical validation. Having said this, we have tried as far as possible to follow a structured diagnostic approach. It should be noted that in this cross-sectional analysis we had no external criterion for validity but rather have used internal social, demographic, lifestyle, personality and clinical variables.

These data are not suitable for estimating population prevalence of exposures or outcomes due to the many selection factors that are likely to be operating. However, the large sample size has resulted in a widely heterogeneous sample which is appropriate for etiologic analyses. Our analyses suggest that the criteria used here for identifying affective disorders are suitable for use in etiological analyses.

The main disadvantage of these data is the self-report nature of the assessments and the need for participants to recall past symptoms and experiences. It is possible that some participants may have under-reported their experience of mood symptoms, especially those with more severe depressive disorders [38]. Although the social stigma relating to mental health disorders such as depression has improved in recent years, stigma remains an issue for many people and may have contributed to some under-reporting of depressive or manic features. Another limitation is that we were not able to assess a wider range of factors which might contribute to some of the differences observed between groups.

This is a large cohort with considerable detail on a wide range of demographic, health and cognitive variables. In keeping with contemporary population cohort studies, the overall recruitment rate was low at around 5.5%. Participants are unlikely to be representative of the general population in terms of age, sex, ethnicity and socioeconomic deprivation and there is likely to have been recruitment bias in favour of a more motivated and relatively highly educated group of individuals [39]. Furthermore, our lifetime estimates of 1.3% for probable bipolar disorder and 25.8% for probable major depression could be considered high given that the low participation rate and the likelihood that depression would be more common among non-participants [40]. Nevertheless, our prevalence estimates are comparable to those reported in other population studies.

## Conclusions

This analysis of features of major depression and bipolar disorder within a large sample of the UK Biobank cohort attempted to quantify the lifetime prevalence of clinically meaningful mood disorders and provide evidence towards validation of our proposed diagnostic criteria for probable major depression and probable bipolar disorder. Our findings provide initial support for these criteria and will lead on to more detailed work on the relationship of depressive and manic symptoms to a range of health outcomes within the cohort. They are also likely to prove useful as a framework for a wide range of future genetic and non-genetic studies.

## Supporting Information

Appendix S1 Unique Data Identifier (UDI) codes
(DOCX)
