# Supplementary material for: Prevalence and Characteristics of Probable Major Depression and Bipolar Disorder within UK Biobank: Cross-Sectional Study of 172,751 Participants
Source: PLoS One. 2013 Nov 25;8(11):e75362. doi: 10.1371/journal.pone.0075362 (PMC3839907; doi:10.1371/journal.pone.0075362)
Supplement: Appendix S1 — Unique Data Identifier (UDI) codes (DOCX) [file pone.0075362.s001.docx]

**Appendix. Unique Data Identifier (UDI) codes**

**Probable bipolar disorder (type I):**

Either: 4642 Ever manic/hyper 2 days or 4653 Ever irritable/argumentative for 2 days, plus

At least 3 from 6156.01 (more active), 6156.02 (more talkative), 6156.03 (needed less sleep), and 6156.04 (more creative/more ideas), plus

5663 Duration of a week or more, plus

5674 needed treatment or caused problems at work

**Probable bipolar disorder (type II):**

Either: 4642 Ever manic/hyper 2 days or 4653 Ever irritable/argumentative for 2 days, plus

At least 3 from 6156.01 (more active), 6156.02 (more talkative), 6156.03 (needed less sleep), and 6156.04 (more creative/more ideas), plus

5663 Duration of a week or more

**Single probable episode of major depression:**

EITHER:

4598 Ever depressed/down for a whole week, plus

4609 At least two weeks duration, plus

4620 Only one episode, plus

2090 Ever seen a GP or 2100 a psychiatrist for nerves, anxiety, depression

OR:

4631 Ever anhedonic (unenthusiasm/uninterest) for a whole week, plus

5375 At least two weeks, plus

5386 Only one episode, plus

2090 Ever seen a GP or 2100 a psychiatrist for nerves, anxiety, depression

**Probable recurrent major depression (moderate):**

EITHER:

4598 Ever depressed/down for a whole week, plus

4609 At least two weeks duration, plus

4620 At least two episodes, plus

2090 Ever seen a GP (but not a psychiatrist) for nerves, anxiety, depression

OR:

4631 Ever anhedonic (unenthusiasm/uninterest) for a whole week, plus

5375 At least two weeks, plus

5386 At least two episodes, plus

2090 Ever seen a GP (but not a psychiatrist) for nerves, anxiety, depression

**Probable recurrent major depression (severe):**

EITHER:

4598 Ever depressed/down for a whole week, plus

4609 At least two weeks duration, plus

4620 At least two episodes, plus

2100 Ever seen a psychiatrist for nerves, anxiety, depression

OR:

4631 Ever anhedonic (unenthusiasm/uninterest) for a whole week, plus

5375 At least two weeks, plus

5386 At least two episodes, plus

2100 Ever seen a psychiatrist for nerves, anxiety, depression
